# Putting the “Return” Back in the Inhibition of Return Effect in Working Memory

**DOI:** 10.5334/joc.401

**Published:** 2024-10-01

**Authors:** Caro Hautekiet, Naomi Langerock, Evie Vergauwe

**Affiliations:** 1 University of Geneva, Switzerland

**Keywords:** working memory, attention, inhibition, focus of attention, perception

## Abstract

The inhibition of return effect in perception refers to the observation that one is slower to re-attend a location that was attended right before, compared to a location that was not attended right before. Johnson et al. (2013, Psych. Sc., 24, 1104–1112, doi:10.1177/0956797612466414) observed a similar inhibitory effect for an attended item in working memory, which the authors referred to as an *inhibition-of-return-like effect*. However, testing an inhibition of *return* effect requires attention to be disengaged from the attended item, before testing whether participants are slower to *return* to said item. This was assumed but not experimentally manipulated in the paradigm by Johnson and colleagues. In the current study, we investigated whether an inhibition of return effect can be observed in working memory when attention is experimentally disengaged from the attended item before measuring whether responses are slower for the item in question. Participants were indeed slower to respond to a memory probe that matched the item that was attended right before, compared to a memory probe that matched the item that was not attended right before. Thus, our test with more experimental control did result in an inhibition of return effect in working memory.

Using attention, one can select and prioritize a specific element among many (e.g., Chun et al., 2011; Desimone & Duncan, 1995). Attention can operate externally, directed toward information in the environment, or internally, directed toward information held in mind (e.g., Chun et al., 2011; van Ede & Nobre, 2023). When attention is focused on information in the environment, this information is assumed to reside in the external focus of attention (e.g., Eriksen & St. James, 1986; Treisman & Gormican, 1988). When attention operates on information in mind, attention is assumed to operate on information held in working memory (the limited-capacity system that temporarily holds information accessible and available, Baddeley & Hitch, 1974). When attention is focused on information within working memory, this information is assumed to reside in the internal focus of attention (e.g., Cowan, 1995; Garavan, 1998; Oberauer, 2002). Currently, it remains unclear whether external and internal attention should be considered as separate entities (e.g., Chun et al., 2011; Tas et al., 2016) or whether they are intimately linked and rely on the same set of cognitive resources (e.g., Awh et al., 2006; Kiyonaga & Egner, 2013, 2014a).

In 2013, Johnson et al. tested whether the well-known inhibition of return (IOR) effect in external attention (Posner & Cohen, 1984) can be observed for internal attention, and concluded that an IOR-like effect exists indeed in working memory. This finding has been taken as support for a close link between working memory and perception, with a close relationship between internal and external attention (e.g., Chen et al., 2017; Gao et al., 2022; Kiyonaga & Egner, 2016). However, we believe there is at least one important conceptual difference between the original paradigm of Posner and Cohen and that of Johnson and colleagues that could complicate the likening of the working memory effect to the classical observation in perception. In what follows, we first explain this difference, before describing how we investigated whether an IOR effect can be observed in working memory while remedying the identified conceptual difference.

## Inhibition of return in perception

**Figure 1:**
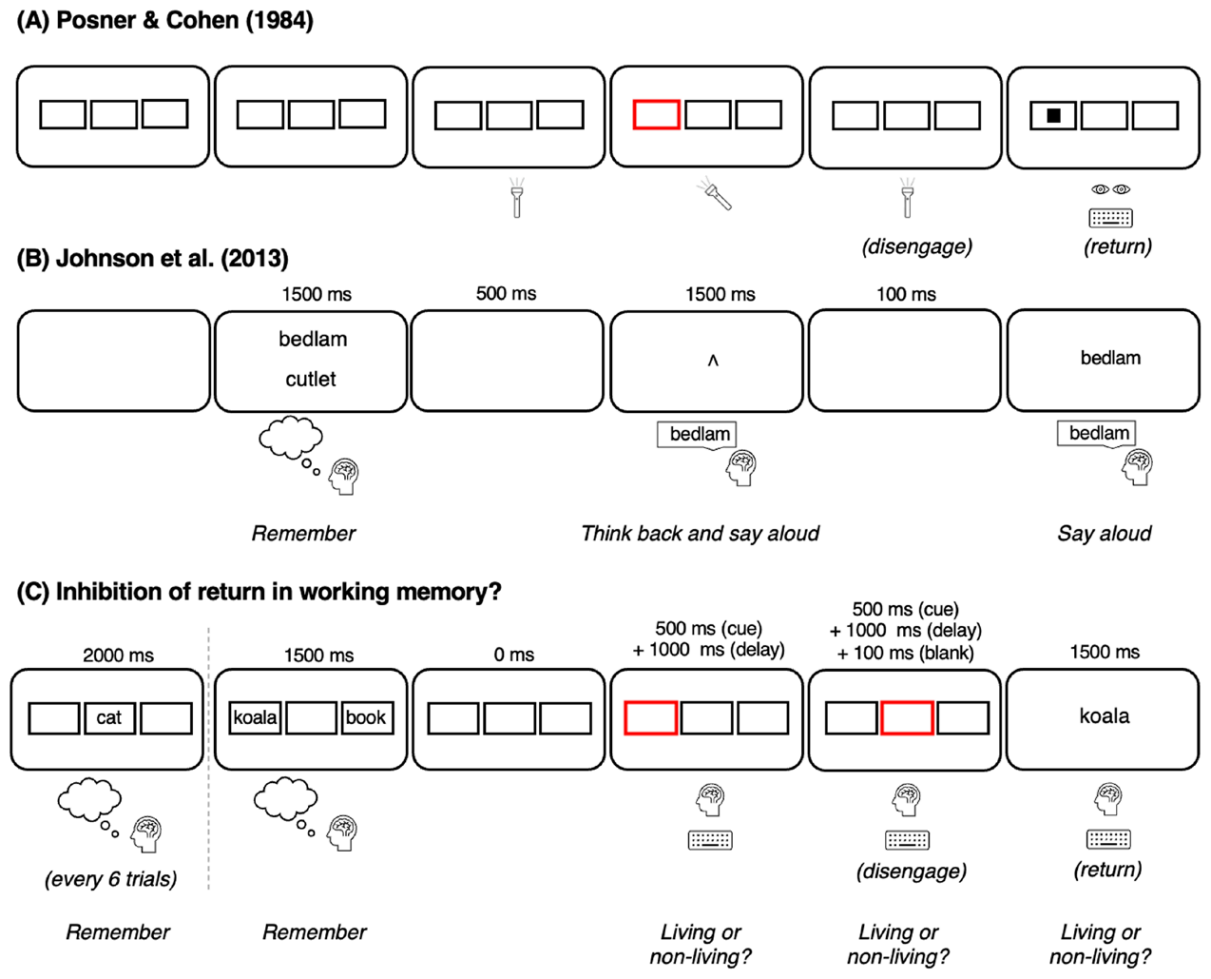
Task procedure (A) Posner and Cohen, 1984, (B) Johnson et al., 2013, and (C) The Current Task. *Note*. Panel A is reproduced and adapted based on “Components of Visual Orienting”, Posner, M. I, and Cohen, Y. (1984), *Attention and Performance, 32*, p. 531–556, Figure 32.1. Panel B is reproduced and adapted based on “Foraging for Thought: An Inhibition-of-Return-Like Effect Resulting From Directing Attention Within Working Memory”, Johnson, M. R, et al. (2013), *Psychological Science, 24* (7), p. 1104–1112, Figure 1a.

In perception research, the IOR effect was first shown by Posner and Cohen (1984). In their task, three rectangles were horizontally presented on screen (Figure 1A). Participants were instructed to keep their eyes fixated on the center of the screen (controlled by eye-tracking). At the beginning of the trial, a cue was presented for 150 ms (brightening of one of the outer rectangles), attracting attention to this location. A probe was presented in the center of one of the rectangles 0–500 ms after the cue. Participants had to press a key as fast as possible upon probe detection. The probe was presented most often in the central rectangle (60% of the trials), motivating participants to refocus their attention centrally after the cue. Presenting the probe in the cued location resulted in faster responses relative to the uncued location when the delay between the cue and probe onset was short (max. 150 ms). However, when the delay was longer (min. 300 ms), presenting the probe in the cued location resulted in slower responses relative to the uncued location. Thus, responses were particularly slow for probes presented in the location that had been the object of the external focus of attention right before. This effect is referred to as the IOR effect in perception.

According to Posner et al. (1985), the mechanism underlying this effect is the capture of attention to the cued location and the following inhibition to return attention to that same location, after attention has been withdrawn from it. The IOR effect is assumed to operate at different stages of processing to discourage orienting to the just-attended location (see Lupiáñez et al., 2007 for a review). This discouragement would come from the evolutionary idea that one should not look for requisites (e.g., food) in a location where one has already looked (see Klein, 2000 and Wang & Klein, 2010, for reviews).

## Inhibition of return in working memory

In working memory research, Johnson and colleagues (2013) presented participants with two to-be-memorized words (Figure 1B). Next, a refreshing cue was presented; participants were instructed to *refresh* the indicated word and say it aloud. Refreshing consists of thinking back to a working memory representation by bringing it into the internal focus of attention (e.g., Barrouillet et al., 2007; Johnson, 1992; Vergauwe & Cowan, 2014, 2015). After, a probe was presented which participants had to read aloud as fast and accurately as possible. The probe could be the refreshed, the unrefreshed, or a novel word. Participants were slower to respond (read) when the probe corresponded to the refreshed word compared to the unrefreshed word. Thus, participants appeared slower to respond to a target when it had been brought into the internal focus of attention right before, compared to another item in working memory, outside the internal focus of attention (see replications by Higgins et al., 2020; Langerock et al., 2021; Lintz et al., 2023). Johnson and colleagues likened this observation in working memory to the well-known IOR effect in perception (see Klein, 2000; Lupiáñez et al., 2007 for reviews) because, in both the perceptual task (Posner & Cohen, 1984) and the working memory task (Johnson et al., 2013), responses to the probe are slower when the probe corresponds to the just-attended location (external) or to the just-attended item (internal).

However, when comparing between the original studies in working memory and in perception, we noticed a potentially important difference between the two paradigms (see Figure 1A and 1B). In Posner and Cohen’s task, participants had a clear incentive to return attention to the center after the cue, because targets were more often presented centrally (resulting in overall faster responses to the central locations compared to the peripheral locations). Later on, many studies used a central cue to exogenously reorient attention to the center after a peripheral cue rather than depend on endogenous control (e.g., MacPherson et al., 2003; Prime et al., 2006). In this type of task, attention can be assumed to be removed from the cued location when the probe appears, allowing assessment of what happens when the focus of attention returns to the cued location. In the paradigm of Johnson and colleagues, there was no such ‘central’ element for participants to attend in between the cue and probe, and thus, in Johnson et al.’s task, it is conceivable that the focus of attention lingers on the cued item. If so, attention might not have been disengaged from the cued item when the probe appears, preventing assessment of what happens when the focus of attention needs to return to the cued item, because the focus of attention might not have been removed from the cued item in the first place. Therefore, Johnson et al.’s effect might not reflect an inhibition of *return* effect in working memory. Moreover, a conceptual replication of the study by Johnson et al. containing six experiments (Hautekiet et al., 2023) did not show any IOR in working memory, casting hence doubt on the IOR interpretation of the results of Johnson et al.’s study. Still, as it is not clear what participants attend to in between the cue and the probe in their task, one could argue that what was observed in their study is indeed IOR. The goal of the present study is to offer a more direct test of an IOR effect in working memory by experimentally disengaging attention away from the cued item. If there is indeed such effect in working memory, this would support the idea of a close link between working memory and perception, with a close relationship between internal and external attention (e.g., Chen et al., 2017; Gao et al., 2022; Kiyonaga & Egner, 2016).

We designed a working memory paradigm that conceptually aligns more closely with the task of Posner and Cohen (1984), by experimentally disengaging the focus of attention from the refreshed item before testing whether participants are slower to return to this item. This way, the refreshed item is no longer the object of focused attention when the probe appears. Specifically, after the refreshing cue, participants were cued to think back to a memorized central item and thus, the focus of attention had to switch away from the refreshed item to the central item before the probe is presented (see Supplementary Materials for a detailed comparison between Johnson et al.’s task and the current study).

The current study was preregistered (see https://osf.io/kxdtv) and the data and analysis script are publicly available on OSF (see https://osf.io/dj7at/).

## Methods

### Participants & Design

We started with 30 participants and used Bayesian sequential hypothesis testing until we obtained a BF of 10 for or against our main hypothesis (after data exclusion), measuring an inhibitory effect, or until max. 60 participants, whichever came first. In total, 60 participants (43 females, 17 males; mean age = 22.02 years) from the University of Geneva took part in the experiment in exchange for course credits. All participants signed an informed consent before participating and the experiment was approved by the ethical commission board at the University of Geneva. The experiment had a 3 (Probetype: refreshed, unrefreshed, or novel) within-subject factorial design. Exclusion criteria were defined based on the first 10 data files as we aimed to be as strict as possible while preserving as much data as possible^1^ (see Supplementary Materials for more information). Participants were included in the data set if they had (1) more than 55% valid trials for the refreshing cue, central cue,^2^ and probe and (2) more than 55% valid trials for the catch trials. A valid trial corresponds to a correct response and reaction time (RT) larger than 150 ms. The final data set consisted of 45 participants (33 female, 12 male; mean age = 22.04 years).

### Procedure

The experiment was created using Psychopy^3^ (v3.2.4; Peirce et al., 2019). Information about the wordlists can be found in the Supplementary Materials. Participants were tested individually, seated at a comfortable distance from the screen.

Each series of six trials started with a to-be-memorized word presented in the middle rectangle for 2000 ms (i.e., the central word; see Figure 1C). One trial consisted of two to-be-memorized words simultaneously presented in the outermost rectangles for 1500 ms. Afterward, the border of one of the outermost rectangles turned red for 500 ms (i.e., the refreshing cue), cuing participants to refresh (i.e., think back to) the previously-presented word and judge if the word presented in that location corresponds to something living (e.g., human being, animals, and vegetables) or non-living (e.g., tools, buildings, and furniture) by pressing one of two keys (“b” and “c”, respectively). This was followed by a 1000 ms presentation of the empty squares during which response registration continued. Next, the border of the middle rectangle turned red (i.e., central cue) and participants were instructed to think back to the previously presented central word and judge whether this was living or non-living by pressing one of two keys (“b” and “c” respectively). This was followed by the presentation of the empty squares for 1000 ms during which response registration continued. After a 100 ms blank screen, a word probe was centrally presented for 1500 ms. Participants were instructed to judge if the presented probe corresponded to a living or non-living item (by pressing “b” or “c”, respectively). Responses for the probe were registered during probe presentation. The presented probe corresponded to the refreshed, the unrefreshed, or a novel word. The central word was never presented as a probe. There was an inter-trial interval of 500 ms. After six consecutive trials, a screen was presented with “New series” (in French). Participants had to press the space key to continue.

To prevent response-based encoding of the words and encourage content-based encoding in working memory instead, we inserted catch trials. A catch trial was the same as a regular trial, apart from the fact that either the refreshing or the central cue was replaced by an image displayed inside the rectangle that would have been cued. In these catch trials, participants were instructed to respond if the image corresponded to the previously presented word or not (by pressing “b” or “c”, respectively). In this way, participants could not simply encode the animacy response.^4^ One third of the trials were catch trials.

In total, there were 252 trials, of which 84 were catch trials which were equally divided between the 3 blocks of the experiment. Within the regular trials, there was an equal distribution of the probe type: 56 refreshed probes, 56 unrefreshed probes, and 56 novel probes (see Supplementary Materials for more details on trial division and practice).

## Results

Analysis was done in R (R Core Team, 2023; v4.3.1) using the BayesFactor package (Morey & Rouder, 2018) with default settings. Performance on catch trials was well-above chance level (84%). Additionally, for the experimental trials, overall accuracy for responses to the refreshing cue, the central cue and the probe was well-above chance level (90%, 86% and 84%, respectively). Overall, this suggests that participants did indeed switch their attention between the different items (see Supplementary Materials for detailed accuracy results). Next, all catch trials were excluded and analyses were done for valid trials only, meaning that the trial had to consist of a correct response to (1) the refreshing cue, (2) the central cue, and (3) the probe to be included in the final dataset (32% of trials excluded).

**Figure 2:**
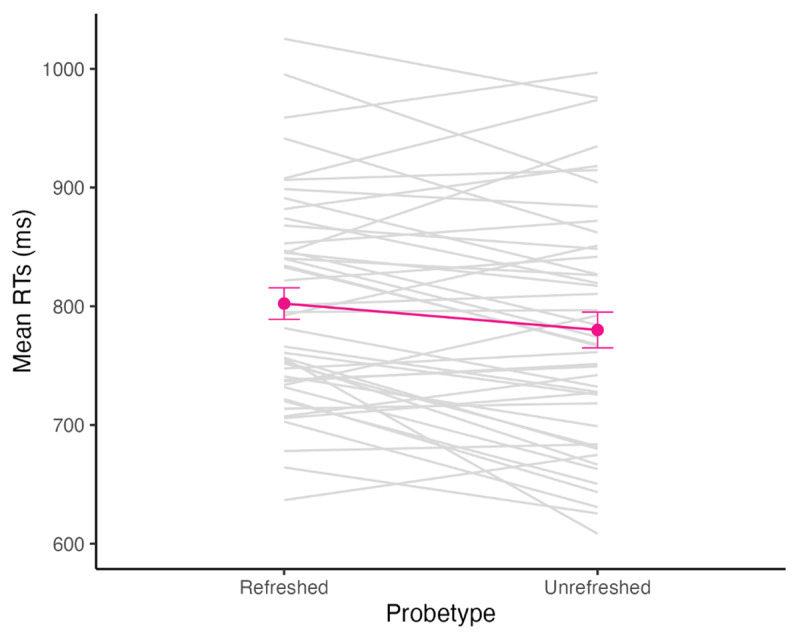
Mean RT (in ms) to the refreshed and unrefreshed probes presented with SE bars (in pink) and individual scores (in grey).

As preregistered, we verified whether RTs were faster for memorized information (refreshed or unrefreshed; M = 792 ms, *SE* = 14 ms) than novel information (M = 823 ms, *SE* = 14 ms) using a Bayesian paired one-sided t-test. This was indeed the case (BF_10_ = 100.91). Next, we ran a Bayesian repeated measures ANOVA of RTs with Probetype (refreshed vs. unrefreshed) as a within-subjects variable. The best model included the main effect of Probetype and was 7 times better than the null model. We then tested more directly for an inhibitory effect (RTs refreshed > RTs unrefreshed) and found strong evidence in favor of this effect (BF_10_ = 12.27). As can be seen in Figure 2, participants were slower to respond to the refreshed probe (M = 802, SE = 13 ms) compared to the unrefreshed probe (M = 780, SE = 15 ms).

## Discussion

Participants were slower to respond to an item that had been in the focus of attention right before (but that was no longer there),^5^ compared to another item in working memory that had not been in the focus of attention right before. Thus, like the findings in perception, we found slower responses when the focus of attention had to return to a recently attended element in working memory.^6^ To our knowledge, this is the first time an inhibition of *return* effect has been observed in working memory using an experimental manipulation ensuring that the focus of attention needs to return to a specific working memory representation. Future research could examine further whether this effect emerges because of suppression of the item itself or the process of re-orienting attention towards the item.

Johnson et al. (2013) did observe a similar pattern (see also replications by Higgins et al., 2020; Langerock et al., 2021; Lintz et al., 2023). However, in their task, there was no experimental manipulation ensuring that the focus of attention was removed from the cued item before the probe appeared. While it is theoretically possible that participants spontaneously switch their attention away from the cued item, there was no specific motivation for participants to do so, and there are at least two reasons why this explanation is unlikely. Firstly, several studies have shown that attention often lingers on a specific item in working memory instead of spontaneously switching away to other items in working memory (e.g., LaRocque et al., 2013; Lewis-Peacock et al., 2012; Vergauwe et al., 2016, 2018). Secondly, it is difficult to argue why participants would spontaneously switch their attention away from the cued item in the task by Johnson et al. (2013), but not in another, highly similar task set-up (see Hautekiet et al., 2023). Therefore, while it remains a possibility, we believe it is rather unlikely that attention was actually disengaged from the attended item in Johnson et al.’s study (see Hautekiet et al., 2023 for more details). As a result, the findings of Johnson et al. might reflect an inhibitory effect other than an inhibition of *return*.

Specifically, the response pattern observed by Johnson et al. (2013) suggests that the item currently residing in the focus of attention is inhibited and therefore, less accessible than other information in working memory. This contradicts with the heightened accessibility that is typically assumed and observed for information in the focus of attention (e.g., Basak & Verhaeghen, 2011; Griffin & Nobre, 2003; Oberauer, 2002; Vergauwe & Langerock, 2017). In a recent study, we found that this type of inhibition is limited to very specific task situations and is most likely unrelated to the functioning of the focus of attention. Specifically, we examined the time course of the effect and the potential role of response inhibition. The results mostly showed a facilitative effect for the refreshed item, but this facilitative effect disappeared when a double oral response was required (to the refreshing cue and the probe). This suggests that there might be some involvement of response inhibition in observing the inhibitory effect^7^ (see Hautekiet et al., 2023, for more details). Overall, it seems that the accessibility of information in the focus of attention is heightened by default, but very specific task situations can mask this heightened accessibility and can even make it seem as if the accessibility is reduced (such as the task used by Johnson et al., 2013). Still, it remains possible that participants spontaneously switched attention away from the cued item in the study by Johnson et al. (2013), and thus, that they indeed observed IOR. In this case, our study still has value as it provides a more direct test of an IOR effect in working memory because attention is experimentally disengaged from the cued item rather than assuming that this happens spontaneously. Future research could examine the differences between the inhibitory effects observed here and in Johnson et al. (2013).

The current observation of IOR in working memory demonstrates that effects that have been shown in the perceptual domain can be found in the working memory domain, supporting the notion that perception and working memory are closely related, with a close relationship between internal and external attention. Similarly, Kiyonaga and Egner (2014b) observed a working memory Stroop effect analogous to the well-known Stroop effect in perception (Stroop, 1935). This demonstrates that representations in working memory can interfere with task performance in a similar manner as items that are externally attended. Similarly, we observed that attention-based inhibition can be observed, regardless of whether attention is turned outwards, to the environment, or inwards, within working memory. Together, these types of studies demonstrate a close relationship between internal and external attention (e.g., Awh et al., 2006; Kiyonaga & Egner, 2013, 2014a; Tsubomi et al., 2013).

## Data Accessiblity statement

The data and analysis script are available on the Open Science Framework: https://osf.io/dj7at/.

## Additional File

The additional file for this article can be found as follows:

10.5334/joc.401.s1 Supplementary materials. The supplementary materials contain more information on the materials, trial division, practice trials, data exclusion criteria, accuracy results, exploratory analyses, a detailed comparison between the task by Johnson et al. (2013) and the current task, as well as some additional plots.

## Footnotes

All considered exclusion criteria levels are included in the preregistration. Based on the first 10 participants, one level was selected and included as exclusion criteria in the preregistration. The results of the critical t-test are provided for each exclusion criteria levels in the Supplementary Materials. This demonstrates that the conclusion remains the same regardless of the selected criteria.

The central cue/item was referred to as the ‘neutral’ cue/item in the preregistration.

The experiment was created to run online using Pavlovia and thus, it was executed using the online version.

For example, if the central word was ‘whale’, response-based encoding would lead to the maintenance of “press b” in WM (because the word refers to a living item). To avoid this and encourage content-based encoding of the word, we included catch trials in which an image was presented for which participants had to judge whether it matched the memorized central (or refreshed) word or not (e.g., in our example, if the image was a whale, the answer would be “yes”; if the image was not a whale, the answer would be “no”). Thus, because on 1/3 of the trials participants would need to know what word had been presented, we assumed they would refrain from simply memorizing “press b” instead of memorizing the actual word.

Previous studies have shown that using a refreshing cue brings the refreshed item in a privileged state within working memory where it benefits from heightened accessibility and improved memory performance in comparison to the unrefreshed items (e.g., Hautekiet et al., 2023; Lintz & Johnson, 2021; Souza et al., 2015, 2018; Vergauwe & Langerock, 2017). Therefore, in this type of task set-up, we assume that only the refreshed item is residing in the focus of attention.

One could argue that this pattern reflects the unrefreshed item residing in the focus of attention rather than active inhibition of the refreshed item. We consider this unlikely for at least three reasons: (1) this would mean that almost all participants consistently behaved in the same way by spontaneously bringing the unrefreshed item into the focus of attention after having returned their attention to the neutral item, without any incentive to focus on the unrefreshed item; (2) previous studies have demonstrated that attention typically lingers on a specific item rather than spontaneously switch away from it (e.g., LaRocque et al., 2013; Lewis-Peacock et al., 2012; Vergauwe et al., 2018; Vergauwe & Langerock, 2022; but see Valentini et al., 2022); and (3) if the unrefreshed item would reside in the focus of attention, we would expect better and faster responses for this item compared to another item in working memory (see Souza & Oberauer, 2016 for a review). While we did observe faster responses for the unrefreshed item, memory performance was still better for the refreshed item compared to the unrefreshed item. This suggests that the unrefreshed item was, most likely, not in the focus of attention when the memory probe was presented. Still, future studies should further investigate this possibility.

It should be noted that this explanation might not apply to Experiment 3 by Johnson et al. (2013) in which visual materials were used and a key press response was required to the probe.

## Author Information

Caro Hautekiet is now at the University of Zurich. The body of the work was completed when Caro Hautekiet was at the University of Geneva.
