# Supplementary material for: Putting the “Return” Back in the Inhibition of Return Effect in Working Memory
Source: J Cogn. 2024 Oct 1;7(1):70. doi: 10.5334/joc.401 (PMC11451542; doi:10.5334/joc.401)
Supplement: Supplementary materials. — The supplementary materials contain more information on the materials, trial division, practice trials, data exclusion criteria, accuracy results, exploratory analyses, a detailed comparison between the task by Johnson et al. (2013) and the current task, as well as some additional plots. [file joc-7-1-401-s1.pdf]

Supplementary materials

1. Supplementary information method section

1.1 Materials

For this experiment, a wordlist of 290 words was derived based on the study of Taikh et al. (2015). The corresponding images originated from the above-mentioned study augmented with the original dataset of Snodgrass & Vanderwart (1980). However, in comparison to these studies, we had a French-speaking sample. Therefore, all words were translated in French using the back-translation technique[1]. After excluding less common and ambiguous words, we were left with 269 words. Additionally, we decided to have a maximal word length of eight letters, thus, this left us with 225 words. In our paradigm, 210 words were necessary for the experimental trials (i.e., 105 living and 105 non-living items) and another 45 words were needed for the practice trials (i.e., three sets of 15 words). Therefore, 210 words were randomly selected from the obtained set of 225 words for the experimental trials. The remaining 15 words were used for the practice trials. Additionally, as a total of 45 words were needed for the practice, we selected a set of 15 words from the excluded words, that were still common or unambiguous enough to be used for practice, and a set of 15 nine-letter words. Words were counterbalanced across conditions and thus, their characteristics (e.g., word length) cannot explain the differences in experimental conditions.

1.2 Trial division and practice trials

In total, participants were presented with 252 trials divided in 3 blocks of 84 trials. Within the 252 trials, there were 84 catch trials and 168 regular trials. Within the regular trials, there was an equal distribution of the probe type: 56 refreshed probes, 56 unrefreshed probes, and 56 novel probes. The catch trials were equally divided between the 3 blocks (i.e.,

[1] All words were translated from English to French and then translated again from French to English by a native French speaking person.

28 catch trials per block). In this way, catch trials could appear at any given moment throughout the experiment. However, there could be no more than 3 catch trials each within each set of six trials. The item that was tested in the catch trials corresponded to the refreshed item in 28 trials, the unrefreshed item in 28 trials, and to a novel item in 28 trials. Additionally, within each block, in 14 catch trials, the refreshing cue was replaced by an image and the other 14 catch trials, the central cue was replaced by an image. In both cases, the image was correct in 7 of the 14 catch trials (i.e., the image corresponded to the previously presented word). When the image replaced the refreshing cue, the image was presented equally in the left or right square. Each block consisted of 14 sets (i.e., each set consists of a central word and six trials) that were presented in a random order. Additionally, the refreshing cue was presented equally often in the left and right square and the order was randomized within each block. Each set had an equal distribution of two refreshed, two unrefreshed, and two novel probe trials that were presented randomly within each set. The words for each set were matched in number of letters and randomly divided in the different positions, such that a word could be either presented as the central word, as part of the memory set or as a novel probe. Furthermore, we made sure the central word was a living item in 50% of the trials. The same sets were used in each block and the words within each set were again divided randomly in the different positions. However, a word was never used twice as a central word.

Before the start of the experimental trials, the task was explained to the participants, showing a concrete example. Then the participant performed 36 training trials (divided in three sets). In the first practice set, participants were presented with a slowed version of the experiment without catch trials and they received feedback for each step of the trial (6 trials). In the second practice set, participants were presented with the experiment at normal pace, but without catch trials (12 trials). Finally, in the third practice set, participants were

presented with the experiment at normal pace with catch trials (18 trials of which 5 catch trials).

1.3 Task comparison

Table S1
Detailed comparison between the task parameters of Johnson et al.'s task and the current task.

Task Phase	Element	Johnson et al. (2013)	Current task
**Encoding central item**	*Set size*	NA	1
	Presentation time	NA	2000 ms
	Frequency	NA	Every 6 trials
**Encoding memory set**	*Set size*	2	2
	Presentation time	1500 ms	1500 ms
	Delay	500 ms	0 ms
**Refreshing cue**	*Nature*	Arrow to location	Highlighted location
	Presentation time	1500 ms	500 ms
	Instruction	Think back to the indicated word and say it aloud	Think back to the indicated word and judge whether it refers to a living or non-living element
	Response	Oral	Key press
	Post-cue Delay	100 ms	1000 ms
**Central cue**	*Format*	NA	Highlighted location
	Presentation time	NA	500 ms
	Instruction	NA	Think back to the indicated word and judge whether it refers to a living or non-living element
	Response	NA	Key press
	Post-Cue Delay	NA	1000 ms + 100 ms blank
**Memory test**	*Probe*	Word	Word
	Instruction	Read aloud the presented word	Judge whether the presented word refers to a living or non-living element
	Response	Oral	Key press
	Response deadline	None	1500 ms

2. Supplementary information results

2.1 Data exclusion criteria

Prior to the preregistration, data of 10 participants was collected to define the exclusion criteria. Specifically, we defined eight possible exclusion criteria, ranging from more strict to less strict (see ‘Explanation of existing data’, https://osf.io/kxdtv/). Next, we applied these steps to the data of the first 10 participants going from most strict to less strict. The first step that resulted in the inclusion of at least 75% was used as the exclusion criteria in the preregistration (see ‘Data exclusion’, https://osf.io/kxdtv/). Proceeding in this way allowed us to be as strict as possible in terms of the exclusion criteria while still preserving as much data as possible. Following this procedure, criterium 3 from the protocol was preregistered to be applied to the entire dataset. That is, participants had to have more than 55% valid trials (valid trial: correct response and RT > 150 ms) for the refreshing cue, central cue, and probe and more than 55% valid trials for the catch trials to be included in the analysis. This resulted in a final data set of 45 participants.

Table S2

Results of the critical Bayesian paired samples t-test measuring for each of the considered exclusion criteria levels.

Level	Participants were included in the final data set if they reached:	Number of participants included (out of 60)	Proportion	Results Bayesian paired samples t-test: RT refreshed > RT unrefreshed
**1**	> 75% valid trials for refreshing cue AND central cue AND probe, and > 75% valid trials on catch trials	32	53%	$BF_{10} = 6.22$
**2**	> 75% valid trials for refreshing cue AND central cue AND probe, and > 55% valid trials on catch trials.	35	58%	$BF_{10} = 5.32$
**3**	> 55% valid trials for refreshing cue AND central cue AND probe, and > 55% valid trials on catch trials.	45	75%	$BF_{10} = 12.27$
**4**	> 75% valid trials for either or both refreshing cue and central cue and > 75% valid trials for both probe and catch trials individually	33	55%	$BF_{10} = 4.22$
**5**	> 55% valid trials for either or both refreshing cue and central cue and > 75% valid trials for both probe and catch trials individually	33	55%	$BF_{10} = 4.22$
**6**	> 55% valid trials for either or both refreshing cue and central cue and > 55% valid trials for both probe and catch trials individually	48	80%	$BF_{10} = 12.01$
**7**	> 75% valid trials for probe and catch trials individually	33	55%	$BF_{10} = 4.22$
**8**	> 55% valid trials for probe and catch trials individually	48	80%	$BF_{10} = 12.01$

2.2 Accuracy results

Table S3

Accuracy scores for the participants included in the final data set.

		Accuracy
Refreshing cue		0.90
Central cue		0.86
Probe	Refreshed	0.83
	Unrefreshed	0.79
	Novel	0.90
	Overall	0.84
Catch	Refreshing cue	0.88
	Central cue	0.80

2.3 Exploratory analysis of the accuracy data

For this exploratory analysis, we only selected the trials in which a correct response had been made to the refreshing cue as well as to the central cue. We executed a repeated measures BANOVA on accuracy with Probe type (three levels; refreshed, unrefreshed, and novel), using the BayesFactor package in R (Morey & Rouder, 2018) with default settings. The results showed very strong evidence for a main effect of Probe type ($BF_{10} = 4.15 \times 10^{19}$). Descriptively, accuracy was higher for the novel probes than the old probes (whether refreshed or unrefreshed) and accuracy was higher for the refreshed probes compared to the unrefreshed probes (see Figure 3). A Bayesian paired one-sided t-test showed that accuracy was higher for the novel probes than for the refreshed probes ($BF_{10} = 3.24 \times 10^{8}$) and the unrefreshed probes ($BF_{10} = 7.72 \times 10^{13}$). Additionally, a Bayesian paired one-sided t-test showed that, accuracy was higher for the refreshed probes than for the unrefreshed probes ($BF_{10} = 7389$).

One could argue that there is a speed-accuracy trade-off for the refreshed vs. unrefreshed probes. While this is possible, it seems unlikely that participants would

strategically focus on accuracy for the refreshed item (vs. the unrefreshed item) and on quick responding for the unrefreshed item (vs. the refreshed item). Alternatively, a more likely possibility is that accuracy is improved for the refreshed item in comparison to the unrefreshed item because refreshing strengthens the memory representation, even when it is not the most recently attended items (e.g., Souza et al., 2015, 2018; Vergauwe et al., 2023)

Figure S1

Accuracy for the refreshed, unrefreshed, and novel probes presented with SE bars (in pink) and individual scores (in grey).

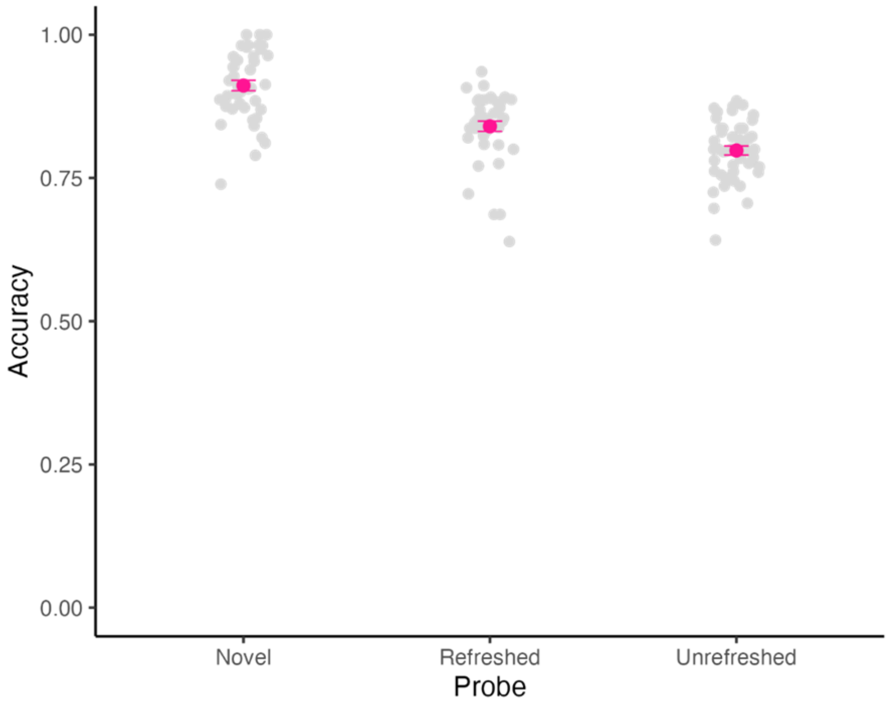

2.4 Additional plots

Figure S2

Mean RT (in ms) for the novel, refreshed, and unrefreshed probes presented with SE bars (in pink) and individual scores (in grey).

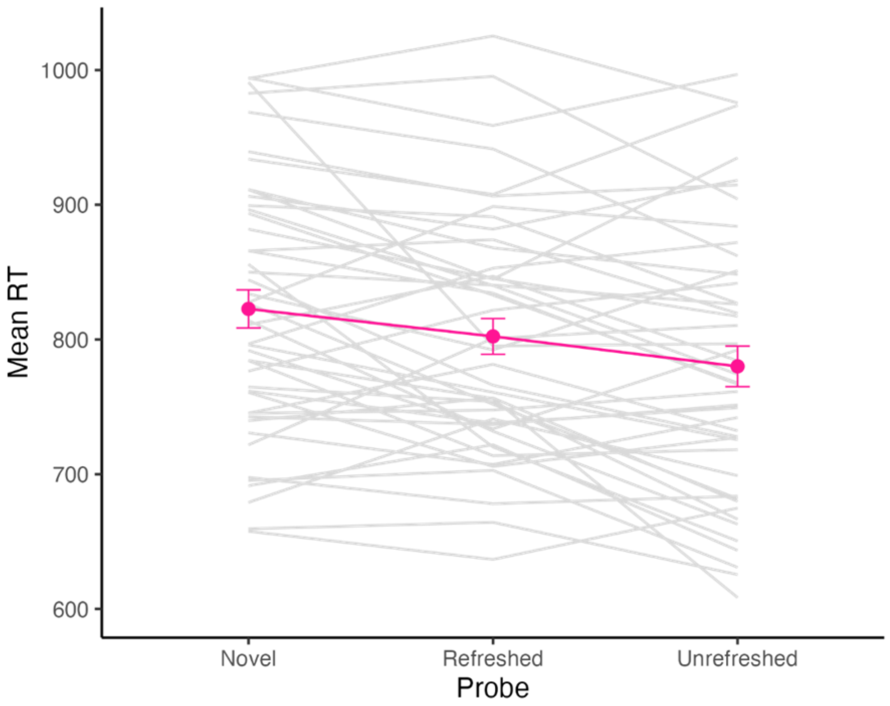

Figure S3

Mean RT (in ms) and accuracy for the refreshing cue and central cue, presented with SE bars (in pink) and individual scores (in grey).

Refreshing cue	Central Cue

(A)

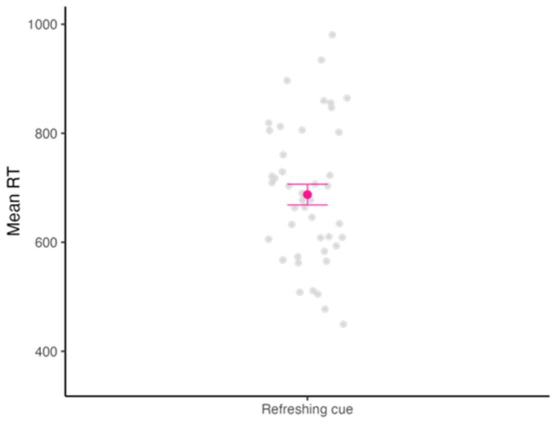

(B)

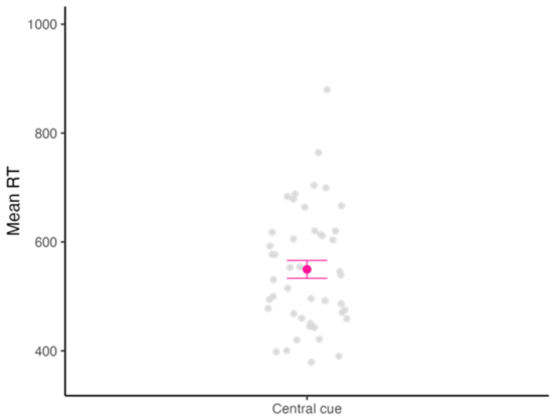

(C)

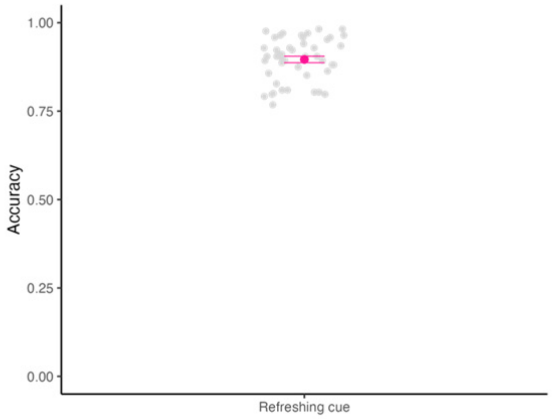

(D)

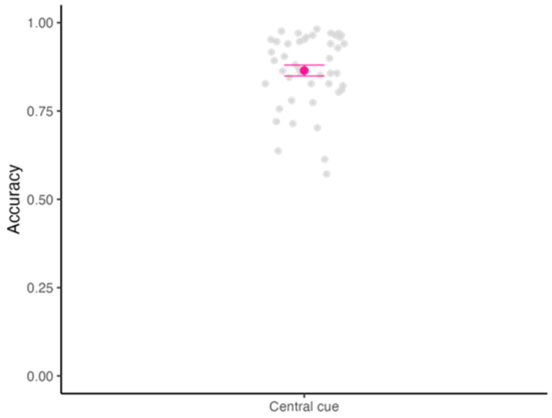

References

Morey, R. D., & Rouder, J. N. (2018). *Baysefactor: Computation of Bayes Factors for Common Designs*.

Snodgrass, J. G., & Vanderwart, M. (1980). A standardized set of 260 pictures: Norms for name agreement, image agreement, familiarity, and visual complexity. *Journal of Experimental Psychology: Human Learning and Memory*, *6*(2), 174–215. https://doi.org/10.1037/0278-7393.6.2.174

Souza, A. S., Rerko, L., & Oberauer, K. (2015). Refreshing memory traces: Thinking of an item improves retrieval from visual working memory. *Annals of the New York Academy of Sciences*, *1339*(1), 20–31. https://doi.org/10.1111/nyas.12603

Souza, A. S., Vergauwe, E., & Oberauer, K. (2018). Where to attend next: Guiding refreshing of visual, spatial, and verbal representations in working memory. *Annals of the New York Academy of Sciences*, *1424*(1), 76–90. https://doi.org/10.1111/nyas.13621

Taikh, A., Hargreaves, I. S., Yap, M. J., & Pexman, P. M. (2015). Semantic classification of pictures and words. *Quarterly Journal of Experimental Psychology*, *68*(8), 1502–1518. https://doi.org/10.1080/17470218.2014.975728

Vergauwe, E., Souza, A. S., Langerock, N., & Oberauer, K. (2023). *The effect of instructed refreshing on working memory: Is the memory boost a function of refreshing frequency or refreshing duration?* PsyArXiv. https://doi.org/10.31234/osf.io/6f4jp
